# Histone Modifications: Decoding the Epigenetic Basis of Economic Traits in Livestock and Poultry

**DOI:** 10.3390/genes17050571

**Published:** 2026-05-18

**Authors:** Yixin Su, Wenze Li, Qi Lv, Rui Su

**Affiliations:** 1 College of Animal Science, Inner Mongolia Agricultural University, Hohhot 010018, China; yixinwink@126.com (Y.S.); 18394317062@163.com (W.L.); lvqi1202@imau.edu.cn (Q.L.); 2 Sino-Arabian Joint Laboratory of Sheep and Goat Germplasm Innovation, Hohhot 010018, China; 3 Inner Mongolia Key Laboratory of Sheep & Goat Genetics Breeding and Reproduction, Hohhot 010018, China

**Keywords:** epigenetics, economic traits, histone modification, livestock and poultry breeding

## Abstract

Economic traits in livestock and poultry arise from the intricate interplay between genetic inheritance and environmental factors, mediated largely by epigenetic regulation. Histone modifications, particularly methylation and acetylation, serve as fundamental epigenetic mechanisms that dynamically remodel chromatin architecture and regulate gene expression in response to developmental and environmental cues. By bridging the gap between static DNA sequences and complex phenotypes, these dynamic marks offer a novel perspective for elucidating trait formation. This review examines the regulatory roles of histone modifications in shaping key economic traits, focusing on skeletal muscle development, fat deposition, and reproductive performance. Furthermore, we highlight two prospective strategies for integrating histone modification data into modern breeding programs: utilizing comprehensive epigenomic maps as novel biomarkers for precision selection, and implementing targeted nutritional regimens to program early phenotypic development. Despite substantial mechanistic advances, critical challenges persist, including high detection costs, inherent tissue specificity, and the necessity to validate transgenerational stability. Looking forward, the integration of multi-omics approaches is anticipated to propel animal breeding beyond traditional genomic selection toward an era of precise epigenomic design.

## 1. Introduction

**Figure 1:**
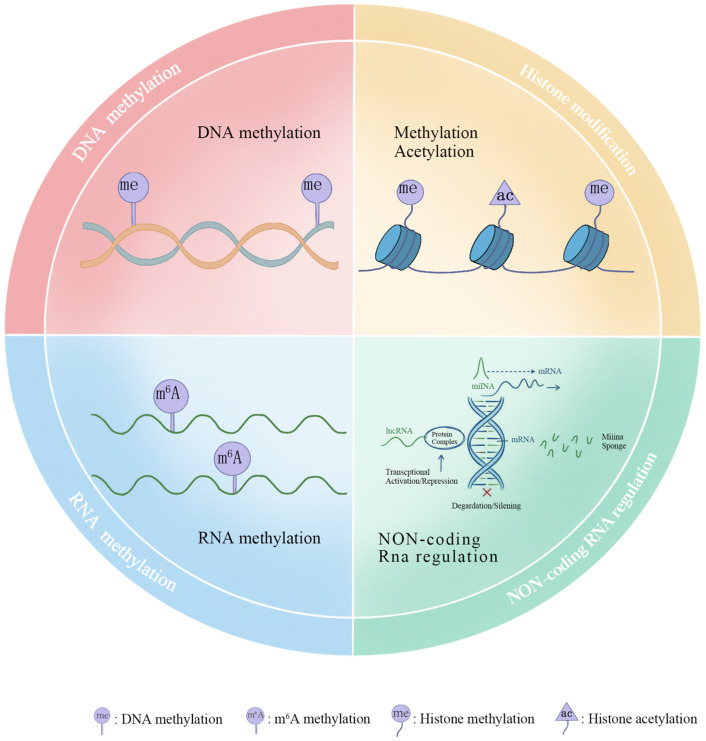
Schematic representation of major epigenetic modifications.

Enhancing the economic traits of livestock and poultry is critical for ensuring global food security and advancing sustainable animal agriculture. The transition from phenotypic selection to genomic selection (GS) has revolutionized traditional breeding by leveraging single nucleotide polymorphism (SNP) data to accelerate genetic gain. However, GS alone cannot fully account for the total phenotypic variance. Challenges such as the “missing heritability,” genotype-by-environment (G × E) interactions, and non-additive genetic effects highlight the limitations of relying solely on DNA sequence information [1]. This gap has driven growing interest in epigenetics, the study of heritable and dynamic changes in gene expression that occur independently of underlying DNA sequence alterations (Figure 1).

Among various epigenetic mechanisms, histone modifications play a central role by forming the biochemical foundation of the “histone code” hypothesis. The N-terminal tails of histones are subject to diverse covalent modifications, including methylation, acetylation, and phosphorylation. These modifications collectively remodel chromatin structure and recruit specific effector proteins to regulate transcription [2,3]. Compared to DNA methylation, histone modifications are highly dynamic and reversible. This plasticity allows them to respond rapidly to environmental and physiological cues, including nutrition, temperature, and pathogen exposure [3]. Consequently, histone modifications serve as a crucial molecular bridge connecting the production environment, the animal genome, and the resulting phenotype.

In the livestock industry, research into histone modifications has progressed from basic mechanistic exploration to the functional annotation of economically important traits. Specific modification patterns, such as the transcriptionally active H3K4 trimethylation (H3K4me3) and H3K27 acetylation (H3K27ac), alongside the repressive H3K27 trimethylation (H3K27me3), have been shown to critically regulate diverse biological processes. These include skeletal muscle development, intramuscular fat deposition, lactation performance, immune response, and stress adaptation across various livestock and poultry species [4,5]. Furthermore, nutritional interventions targeting these epigenetic marks demonstrate significant potential for modulating animal growth and health [6]. Together, these findings suggest that integrating histone modification data could propel modern animal breeding beyond conventional genomic selection into an era of epigenomic-assisted selection and targeted developmental programming.

This article reviews the fundamental mechanisms and potential applications of histone modifications in animal husbandry. We summarize the molecular pathways through which core histone marks regulate key economic traits in livestock and poultry. Furthermore, we outline two practical strategies for incorporating histone data into modern breeding frameworks: utilizing epigenetic signatures as auxiliary selection biomarkers and shaping desirable phenotypes through precision nutritional programming. Finally, we discuss current technical challenges and future perspectives, aiming to provide a theoretical foundation for advancing epigenetic research and its industrial application in animal production.

## 2. The Molecular Machinery of Histone Modifications

**Figure 2:**
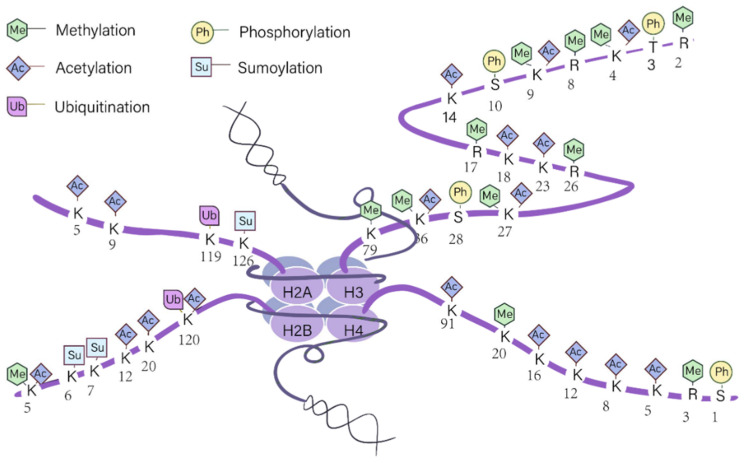
Plot of the histone modification sites.

Histone modifications serve as a fundamental epigenetic mechanism for regulating chromatin architecture. Through reversible covalent labeling of core histone N-terminal tails, these modifications dynamically influence nucleosome structure, chromatin compaction, and transcriptional activity without altering the DNA sequence. This molecular plasticity, often referred to as the “histone code”, endows cells with the capacity to adapt to developmental cues and environmental signals, while maintaining overall genomic stability. Among these, histone methylation and acetylation are the most extensively characterized and have clearly defined roles in regulating economically important traits in livestock and poultry (Table 1). The major epigenetic modification types and their regulatory sites are illustrated in Figure 2.

### 2.1. Histone Methylation

Histone methylation involves the covalent addition of methyl groups to specific lysine or arginine residues. The precise regulation of this process is orchestrated by three classes of functional proteins: “writers” (histone methyltransferases, HMTs), “erasers” (histone demethylases, KDMs), and “readers” (effector proteins with specific recognition domains) [3].

“Writers” (histone methyltransferases) utilize S-adenosylmethionine (SAM) as a methyl donor to deposit specific methyl groups onto histone tails, thereby altering chromatin structure and regulating gene expression. For example, the PRC2 complex establishes the H3K27me3 mark, which generally functions to repress target gene transcription [7]. Conversely, erasers, such as the KDM family, reversibly remove these marks to maintain dynamic equilibrium [8]. Finally, reader proteins decode these chemical signals by selectively binding to specific methylation states and recruiting downstream chromatin remodelers or transcriptional regulators [9].

The dynamic balance between functionally opposing marks is exquisitely illustrated by “bivalent domains”, regions harboring both the activation-associated H3K4me3 and the repression-associated H3K27me3. This unique configuration poises master regulatory genes for rapid activation or repression, acting as a critical epigenetic switch governing early embryonic development, myogenesis, and adipogenesis in farmed animals [10].

### 2.2. Histone Acetylation

Histone acetyltransferases (HATs) catalyze the transfer of acetyl groups from acetyl CoA to lysine residues on histone tails. This neutralizes the positive charge of lysines, thereby reducing the electrostatic attraction between histones and DNA. The consequent chromatin relaxation increases the accessibility of genetic loci to transcription factors and RNA polymerase II. Because HATs require acetyl CoA as an obligate substrate, histone acetylation is directly coupled to cellular energy metabolism. In livestock, variations in nutritional intake and feed efficiency alter intracellular acetyl CoA levels [11]. Such fluctuations can rapidly reshape the acetylation landscape in tissues like muscle, liver, and mammary gland, ultimately modulating transcriptional programs that control growth, lactation, and meat quality.

Histone deacetylases (HDACs) remove acetyl groups from lysines, restoring the positive charge on histones and promoting chromatin compaction. This activity counterbalances acetylation and leads to transcriptional repression. The mammalian HDAC family contains 18 isoforms, which are grouped into four classes based on homology to yeast HDACs. Classes I, II, and IV are zinc dependent and function broadly in epigenetic maintenance. Class III members, known as sirtuins (SIRT1 to SIRT7), are fundamentally different. They require NAD^+^ as an essential cofactor instead of zinc. This requirement ties sirtuin activity tightly to the cellular NAD^+^/NADH ratio, an indicator of energy status and metabolic stress [12,13]. Consequently, sirtuins act as epigenetic sensors that translate fasting, heat stress, or nutrient restriction into changes in deacetylation and gene expression. In livestock and poultry, this mechanism provides a direct molecular link between environmental challenges and phenotypic adaptation [1].

### 2.3. Other Modifications

In addition to methylation and acetylation, several other post-translational modifications of histones have been implicated in regulating economic traits in livestock and poultry. Here, we focus on three representative ones: phosphorylation, ubiquitination, and lactylation.

Histone phosphorylation refers to the reversible addition of a phosphate group to the side chains of serine, threonine, or tyrosine residues on histone tails, primarily catalyzed by protein kinases. This modification introduces a negative charge, which alters chromatin structure and regulates gene transcription. It plays key roles in chromosome condensation during mitosis, DNA damage repair, and cellular signal transduction. The reverse process, dephosphorylation, is mediated by protein phosphatases, which remove phosphate groups and terminate the signalling response. Histone ubiquitination involves the covalent attachment of ubiquitin (a small regulatory protein) to lysine residues on histones, typically catalyzed by E1 (activating), E2 (conjugating), and E3 (ligase) enzymes. Unlike small chemical modifications, ubiquitination adds an entire protein, allowing for diverse regulatory outcomes. The most well-studied forms are monoubiquitination of H2B (H2Bub1) and H2A (H2Aub), which influence transcription elongation and gene silencing, respectively. Deubiquitination is carried out by deubiquitinating enzymes (DUBs), which remove ubiquitin moieties and reverse the modification [14]. Histone lactylation is a novel modification first described in 2019, where a lactyl group is added to lysine residues. It is catalyzed by acyltransferase and provides a direct link between cellular metabolism and epigenetic regulation. Lactylation has been shown to promote gene activation. Delactylation is mediated by class I and II HDAC, which remove lactyl groups and reverse the modification [15].

## 3. Histone Modifications as Drivers of Economic Traits

**Figure 3:**
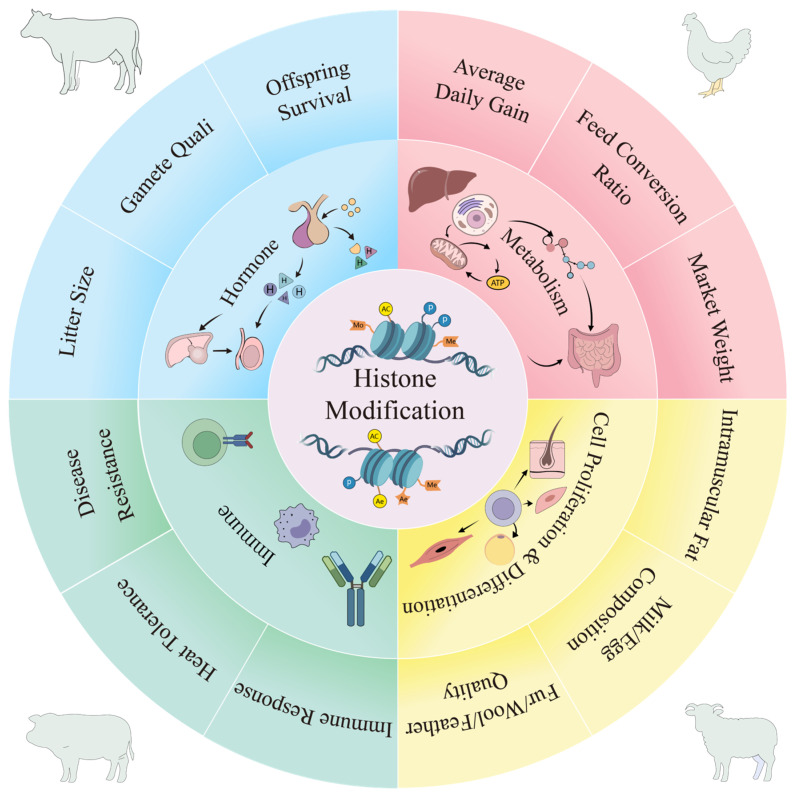
Histone modifications mediate physiological processes to shape economic traits in livestock.

Among diverse histone modification patterns, H3K4me3 and H3K27me3 in histone methylation, as well as H3K9ac and H3K27ac in histone acetylation, have accumulated the most abundant and convincing experimental evidence for regulating growth, reproduction, environmental adaptation and immune-related economic traits in livestock and poultry (Figure 3). By comparison, other types of histone modifications have been preliminarily reported in domestic animals, but systematic functional verification and mechanistic exploration remain relatively insufficient.

### 3.1. Histone Methylation in Economic Traits

Histone methylation, a fundamental epigenetic mechanism, dynamically alters chromatin architecture by adding methyl groups to specific histone residue, thereby activating or repressing gene transcription. Multi-species transcriptomic and chromatin structure annotations in domesticated animals have been continuously refined [16]. Consequently, the key molecular mechanisms of histone methylation in livestock meat production, reproduction, lactation, and stress resistance have been progressively elucidated. 

#### 3.1.1. Muscle Development and Fat Deposition

Histone methylation plays critical roles in skeletal muscle development and fat deposition, two biological processes that collectively determine meat production and quality in livestock.

During skeletal muscle development, the dynamic changes of histone methylation marks strictly regulate the timely expression of myogenic genes, with the repressive mark H3K27me3 and the activating mark H3K4me3 playing core roles. Multi-omics epigenetic analyses have further uncovered histone modification-driven regulatory mechanisms underlying muscle heterosis in sheep, illustrating the vital roles of dynamic histone marks in ovine skeletal muscle growth and phenotypic superiority formation [17]. This H3K27me3-mediated repression mechanism is highly conserved across livestock species. In the late stages of ovine fetal skeletal muscle development, numerous genes related to muscle contraction and development are repressed by the H3K27me3 mark, with targets highly consistent with those in mice [18].

In porcine embryo development, genome-wide H3K27me3 levels continuously increase and are negatively correlated with gene expression. The depletion of H3K27me3 at promoter regions triggers the transcriptional activation of key genes involved in skeletal muscle morphogenesis and myofiber maturation, such as *DES*, *MYL1*, *TNNC1*, and *KLF5* [19]. In vitro validation in porcine satellite cells further demonstrated that global H3K27me3 levels decrease by approximately 50% during differentiation, and the removal of this mark significantly promotes myogenic differentiation by upregulating 139 myogenic genes [20]. Similarly, computational analyses in Nelore beef cattle predict that H3K27me3 significantly affects muscle gene expression and mineral content [21]. Conversely, activating marks is equally indispensable. For instance, during the development of the longissimus dorsi muscle in fetal yaks, H3K4me3 modification plays a crucial role in the early development of muscle satellite cells and the formation of primary myofibers [22].

In addition to global methylation marks, specific histone methylation modifiers have been proven to be directly associated with muscle growth and meat quality traits in livestock. In pigs, the expression of the H3K36 demethylase JHDM1A gradually increases in embryos from day 33 to day 90 of gestation. More importantly, a C224G polymorphism in this gene is significantly associated with average daily gain, drip loss, type II myofiber proportion, shear force, and lactate content, with distinct allele frequency differences observed between pig breeds of distinct growth rates [23]. In poultry, the methyltransferase-like gene *METTL21C* is specifically expressed in skeletal muscle and acts as a key epigenetic modifier to actively promote myogenic differentiation [24].

Regarding fat deposition, histone methylation plays essential roles in regulating lipid metabolism and adipogenesis. In pigs, MAT2A promotes adipogenesis by catalyzing H3K27me3 deposition at the *Wnt10b* locus, thereby repressing the anti-adipogenic Wnt/β-catenin signaling pathway [25]. The spatial and quantitative dynamics of these marks are also crucial; during adipogenic differentiation of porcine mesenchymal stem cells, the repressive marks H3K27me3 and H4K20me3 increase and relocate to the nuclear periphery, while H3K9me3 remains unchanged. Concurrently, the activating marks H4K8ac and H3K9ac increase, whereas H3K4me3 shows no significant quantitative change but preferentially occupies peripheral nuclear regions in mature adipocytes [26]. Beyond cell differentiation, EZH2, the enzyme that catalyzes H3K27me3, regulates adipocyte lipid metabolism independently of adipogenesis, partially through apolipoprotein E [27]. In addition to H3K27me3, the dynamic regulation of other methylation marks is pivotal for fat accumulation. For instance, the histone methyltransferase MLL4 (KMT2D) drives adipocyte differentiation by depositing activating H3K4me1/3 marks at the enhancers of master adipogenic transcription factors, such as PPARγ and C/EBPα [28]. This epigenetic regulation directly impacts on economic traits in ruminants. In cattle, a comprehensive review highlighted those repressive epigenetic mechanisms, including H3K27me3, heavily influence intramuscular fat deposition, which directly affects beef marbling and economic value [29].

#### 3.1.2. Reproduction and Embryonic Development

In male reproduction, several histone methyltransferases regulate spermatogenesis. In goats, EZH2, a histone H3 lysine 27 (H3K27) methyltransferases, is highly expressed in spermatogonial stem cells (SSCs) compared to Leydig and Sertoli cells. Knockdown of EZH2 reduces SSC proliferation and viability, blocks the G2/M phase of the cell cycle, and alters expression of apoptosis-related genes (*CASP3*, *CASP9*, *BAX* up-regulated; *BCL2* down-regulated), along with decreased expression of the SSC marker GDNF and the spermatogenesis-related gene *DAZL*, suggesting that EZH2 plays a pivotal role in SSC self-renewal and that its knockdown impairs spermatogenesis [30]. Another study further demonstrated that miR-101–5p overexpression suppresses goat SSC proliferation and promotes apoptosis by targeting EZH2, reinforcing the critical role of EZH2 in maintaining spermatogenesis [31]. In pigs, SETDB1, a histone methyltransferase catalyzing H3K9 trimethylation, is increasingly expressed during testis development and strongly localized in gonocytes. Knockdown of SETDB1 leads to gonocyte apoptosis and a decrease in H3K27me3 levels, with no significant change in H3K9me3, indicating that SETDB1 contributes to gonocyte survival in pigs through regulation of H3K27me3 rather than H3K9me3 [32]. Comparative analysis revealed increased expressions of MLL5, SETDB1 and SUV420H1, while SETDB2 and EZH2 were decreased in cattle-yak testes at 10 months. Furthermore, significant enrichment of H3K27me3 and H4K20me3 in Sertoli cells, alongside altered H3K4me3, H3K9me1, and H3K9me3 in meiotic chromosomes, highlights the critical involvement of histone methylation in spermatogenic failure and hybrid male sterility [33]. In cattle, H3K27me3 methylation levels in spermatozoa are inversely correlated with bull fertility, with methylation intensity higher than acetylation intensity and significantly differing between high- and low-fertility groups, suggesting that H3K27me3 in sperm may serve as a potential epigenetic marker for male fertility [34].

In female reproduction and early embryogenesis, histone methylation undergoes extensive reprogramming during oocyte maturation and preimplantation development. A genome-wide study profiling H3K4me3, H3K27me3, and H3K27ac in porcine oocytes and preimplantation embryos revealed the existence of unusual broad H3K4me3 domains not only in oocytes but also expanded in pre-ZGA embryos. Notably, broad H3K27me3 and H3K27ac enriched domains were found to be preferably colocalized with broad H3K4me3 domains in pigs, in contrast to their distribution in mice and humans. Functional experiments showed that knocking down KDM5B in pig embryos disrupted cleavage and development, which is consistent with findings in mice. This study also demonstrated that aberrant reprogramming of H3K4me3 and H3K27me3 triggers zygotic genome activation (ZGA) dysregulation in somatic cell nuclear transfer (SCNT) embryos, and that H3K27me3-mediated imprinting does not exist in porcine IVF and SCNT embryos. The similarities observed between porcine and human histone modification dynamics suggest that the pig may serve as a useful model for human embryo research [35]. A multi-species comparative study across humans, cattle, pigs, rats, and mice showed that non-canonical broad H3K4me3 and H3K27me3 domains exist in oocytes of all species except humans. After zygotic genome activation (ZGA), non-canonical H3K4me3 transitions to canonical patterns, likely mediated by the H3K4me3 demethylase KDM5B. Importantly, H3K27me3 is globally erased around ZGA in humans, cattle, and pigs, whereas in rodents it can be inherited to the blastocyst stage, suggesting that H3K27me3-mediated non-canonical genomic imprinting may be specific to rodents [36]. Furthermore, high DNA methylation in bovine and porcine oocytes is strongly correlated with H3K36me2 and H3K36me3, two histone modifications known to mediate DNA methylation during gametogenesis in mice, yet the distribution of H3K36me2 and H3K36me3 in cattle and pig oocytes is remarkably similar, indicating both conservation and species-specificity in the mechanisms of de novo DNA methylation establishment across species [36].

In terms of functional studies, disruption of histone methylation dynamics impairs embryonic development. Knockdown of KDM5B in porcine embryos disrupts cleavage and development, consistent with findings in mice [35]. In bovine embryos, inhibition of *PRAMEY*, a Y-linked gene involved in germ cell formation and fertilization, significantly reduces H3K27me3 methylation at the 8-cell and morula stages while not affecting H3K9me3, underscoring its role in early embryonic epigenetic regulation [37]. In the context of somatic cell nuclear transfer (SCNT) in pigs, excessive H3K9me3 and H3K27me3, but not H3K4me3, were observed in genomic regions with unfaithful embryonic genome activation and donor cell-specific gene silencing. A combination of the H3K9 demethylase KDM4A and GSK126 (an H3K27me3 inhibitor) removed these epigenetic barriers and restored the global transcriptome in SCNT embryos, while TDG was identified as a pig-specific epigenetic regulator that was not reactivated by H3K9me3 and H3K27me3 removal, offering valuable methods to increase cloning efficiency [38]. A comprehensive review further highlighted that histone modifications, including H3K4me3 and H3K27me3, regulate embryo implantation in livestock by modulating key genes such as *HOXA10* and *LIF*, with H3K4me3 enrichment in trophoblast cells positively correlated with embryonic invasion capacity, and the spatiotemporal expression of the H3K27me3 demethylase KDM6B is critical for determining the endometrial “implantation window” [39].

#### 3.1.3. Lactation and Metabolism

Lactation is a highly energy-consuming physiological process, and mammary gland development as well as milk component synthesis are profoundly regulated by epigenetic mechanisms [40,41,42]. Recently, integrative multi-omics analyses have unraveled the complex epigenetic networks within the mammary gland tissue of dairy cattle, providing new genetic insights into milk production traits [43]. Histone methylation not only directly regulates the activation of lactation-related genes but is also deeply involved in the pathogenesis of nutritional and metabolic disorders. For instance, in laying hens with fatty liver hemorrhagic syndrome (FLHS) induced by high-energy and low-protein diets, the epigenetic regulation of H3K27me3 undergoes abnormal alterations, revealing how nutritional imbalance mediates lipid metabolism disorders through the epigenetic layer [44].

#### 3.1.4. Environmental Adaptation and Immunity

Heat stress poses a significant challenge to livestock production, adversely affecting growth, reproduction, and health. Epigenetic mechanisms, including histone modifications, have been increasingly recognized as key mediators of thermal adaptation. A comprehensive review highlighted that heat exposure triggers epigenetic reprogramming involving DNA methylation and histone modifications, which together regulate the transcriptional levels of heat-responsive genes and protect cells from thermal damage [45]. Specifically, histone modifications are dynamically altered during heat stress to modulate the expression of heat shock proteins (HSPs) and other stress-responsive factors [45]. In chickens, thermal manipulation during embryogenesis impacts H3K4me3 and H3K27me3 histone marks in the hypothalamus, suggesting that early-life thermal conditions can induce lasting epigenetic changes that influence thermotolerance later in life [46]. Furthermore, an integrated transcriptome and histone modification analysis revealed that Newcastle disease virus infection under heat stress affects bursa development and proliferation in susceptible chicken lines, demonstrating the complex interplay between thermal stress and immune challenge at the epigenetic level [47].

Histone methylation also plays critical roles in regulating immune responses and disease resistance in livestock. In chickens, genome-wide histone modification analysis revealed distinct patterns between lines resistant and susceptible to Marek’s disease virus (MDV) infection. Specifically, resistant chickens exhibited H3K4me3 enrichment at activated genes associated with immune responses and cell adhesion. Conversely, susceptible chickens showed H3K27me3 patterns targeting genes involved in 5-HT and adrenergic receptor pathways. These contrasting profiles suggest that intrinsic epigenetic mechanisms are key determinants of MDV resistance and susceptibility [48]. In cattle, a study on bovine mastitis resistance to Staphylococcus aureus demonstrated that H3K27me3 exerts a repressive regulatory role on immune- and disease-related genes, with differentially expressed genes negatively regulated by H3K27me3 modification at promoter-proximal regions [49]. Moreover, a recent review explored epigenetic control of immune responses to Mycobacterium bovis infection in cattle. It noted that histone modifications and DNA methylation are dynamically altered by the pathogen, which may ultimately determine infection outcome and diagnostic test sensitivity [50]. In pigs, a study on antiviral signature genes in porcine macrophages found that macrophage activation alters expression of genes encoding DNA/histone methyltransferases, histone deacetylases, and histone demethylases. Epigenetic inhibitors were also shown to suppress PRRSV infection in MARC-145 cells and porcine macrophages [51].

### 3.2. Histone Acetylation in Economic Traits

Histone acetylation, particularly at H3K27ac and H3K9ac, serves as a prominent epigenetic mark associated with open chromatin and transcriptional activation. Mediated by histone acetyltransferases (HATs) and histone deacetylases (HDACs), this dynamic modification orchestrates the interplay between environmental stimuli and gene expression programs. In livestock and poultry, histone acetylation profoundly impacts economic traits including muscle development, embryonic reprogramming, lactation physiology, and environmental adaptation.

#### 3.2.1. Muscle Development and Fat Deposition

In pigs, the transcription factor SATB2 binds to histone deacetylase 4 (HDAC4) and positively regulates skeletal muscle cell proliferation and migration. SATB2 is expressed at higher levels in lean-type pigs than in obese-type pigs, and it enriches pathways involved in skeletal muscle development and chromatin organization. Functional studies have shown that HDAC4 promotes skeletal muscle cell proliferation and migration, and the SATB2-HDAC4 axis mediates chromatin remodeling to influence myogenesis [52]. Additionally, the dietary compound sulforaphane (SFN) enhances porcine satellite cell proliferation by inhibiting HDAC activity, elevating acetylated histone H3 and H4 levels, and increasing H4 acetylation at the SMAD7 promoter [53]. In chickens, HDAC4 knockdown inhibits the proliferation and differentiation of skeletal muscle satellite cells, indicating that HDAC4 plays a positive role in chicken skeletal muscle growth and development [54]. Moreover, HDAC2 has been identified as a functional gene for skeletal muscle development in chickens. Its expression increases significantly during embryonic myoblast hyperplasia and is higher in fast-growing muscles than in slow-growing muscles at 90 days of age, suggesting a role in both pre-hatch and post-hatch muscle development [55]. In cattle, HDAC11 promotes the proliferation of bovine muscle stem cells through the Notch signaling pathway while inhibiting myoblast differentiation by reducing MyoD1 transcription, thereby suppressing muscle regeneration [56]. A comprehensive review has further highlighted the role of histone acetylation and deacetylation in skeletal muscle metabolism. These modifications dynamically regulate gene expression through the coordinated action of HATs and HDACs. They influence key processes including the muscle cell cycle, muscle fiber type conversion, and muscle atrophy [57].

Regarding fat deposition, histone acetylation also plays crucial roles in regulating adipogenesis and lipid accumulation in livestock. In pigs, the novel protein modification lysine 2-hydroxyisobutyrylation (Khib) has been identified as a key regulator of adipogenesis and fat deposition. The Khib levels increase quadratically during adipogenic differentiation of fibro-adipogenic progenitors (FAPs), in parallel with changes in its regulatory enzymes lysine acetyltransferase 5 (KAT5) and HDAC2. KAT5 knockdown inhibits adipogenic differentiation, whereas HDAC2 knockdown enhances it. Moreover, Khib levels are higher in backfat tissues of obese-type pigs (Laiwu pigs) than in lean-type pigs (Duroc pigs), and the expression patterns of KAT5 and HDAC2 correlate with the degree of backfat accumulation [58]. The HDAC inhibitor trichostatin A (TSA) has been shown to enhance adipocyte formation in porcine stromal vascular fraction by nearly 100% and increase the expression of *PPARG* and *CEBPA* mRNAs by 150% and 50%, respectively, further demonstrating the role of histone acetylation in porcine adipogenesis [59]. Histone modification-mediated enhancer activation critically regulates brown fat thermogenesis in sheep. A functionally conserved epigenetic enhancer modulates *PGC1A* transcription and adipose thermogenic programs, providing an important epigenetic basis for fat metabolism and stress adaptation in sheep [60].

#### 3.2.2. Reproduction and Embryonic Development

In male reproduction, the balance between histone acetyltransferases (HATs) and histone deacetylases (HDACs) regulates spermatogenesis. A comprehensive review has highlighted the importance of HATs and HDACs in mammalian spermatogenesis, with particular emphasis on understanding their expression changes during this process [61]. In yaks and cattle-yaks, the testicular mRNA and protein levels of HAT1 were significantly lower in sterile hybrids than in fertile yaks, while HDAC1 protein levels were significantly higher. Consequently, H3K9 acetylation levels in cattle-yak testes were markedly reduced. These results suggest that male sterility in cattle-yaks may be associated with decreased histone acetylation in the testes [62].

In female reproduction, histone acetylation is essential for oocyte meiotic maturation and developmental competence. In pigs, class IIa HDACs play a crucial role in regulating histone deacetylation during oocyte meiosis. Treatment with the class IIa-specific HDAC inhibitor MC1568 increased H4K12 acetylation levels in SCNT one-cell and two-cell embryos, enhanced blastocyst formation rates and cell numbers, and stimulated expression of development-related genes *OCT4*, *CDX2*, *SOX2*, and *NANOG* [63]. Conversely, HDAC11 inhibition in porcine oocytes disrupts meiosis by affecting α-tubulin acetylation, histone modifications, and transcriptome profiles [64]. In cattle, HDAC1 knockdown in oocytes did not affect maturation or cleavage rates, but significantly reduced blastocyst development and increased H3K14 acetylation levels in parthenogenetic embryos [65].

Histone acetylation also undergoes extensive reprogramming during early embryonic development and is critical for zygotic genome activation (ZGA). In pigs, H3K27ac levels gradually decrease from the pronuclear stage to the 8-cell stage, corresponding to the major wave of embryonic genome activation. This is followed by re-acetylation from the morula stage onwards, coinciding with initial cell lineage specification. Similar dynamic patterns were observed in SCNT and parthenogenetic embryos, although H3K27ac levels were slightly lower in late SCNT blastocysts [66]. The transcription factor LEUTX, a key PRD-like activator of porcine ZGA, opens EGA-related genomic regions and establishes histone acetylation by recruiting acetyltransferases p300 and KAT2A. LEUTX overexpression restores ZGA failure and improves preimplantation development and cloning efficiency in porcine cloned embryos [67]. Another transcription factor, TBX3, regulates histone acetylation and plays important roles in ZGA and early embryonic development in pigs. TBX3 knockdown decreased H3K9ac and H3K27ac levels, reduced ZGA gene expression at the four-cell stage, and lowered pluripotency gene expression in blastocysts [68]. Maternal KAT8, an acetyltransferase that specifically catalyzes H4K16 acetylation, is required for porcine preimplantation embryo development. KAT8 mRNA is maternally derived, and its abundance continuously decreases throughout meiotic maturation and preimplantation development [69].

#### 3.2.3. Lactation and Metabolism

In the bovine mammary gland, histone acetylation directly influences lactation performance. Lipopolysaccharid (LPS) increases HDAC activity in a dose-dependent manner, leading to a significant reduction in histone H3 acetylation levels in MAC-T cells. Sodium butyrate, an HDAC inhibitor, effectively reverses this decline and enhances the mRNA expression of lactation-related genes. These findings suggest that LPS impairs lactation by reducing H3 acetylation, and that butyrate can mitigate this adverse effect through HDAC inhibition [70]. The short-chain fatty acids sodium propionate and sodium butyrate also inhibit class I HDAC activity in bovine mammary epithelial cells in a concentration-dependent manner. Sodium butyrate increases acetylation at H3K9/14, H3K18, and H3K27, while sodium propionate increases acetylation at H3K9/14 and H3K18 [71]. Moreover, valine promotes milk synthesis in bovine mammary epithelial cells via the TAS1R1-mTOR-DDX39B signaling pathway. DDX39B directs PKM2 accumulation in the nucleus, which weakens the interaction between HDAC3 and histone H3, thereby increasing H3 acetylation. In vivo, valine-enriched drinking water elevates milk protein and fat expression in mice, confirming that valine enhances milk synthesis through H3 acetylation [72].

Beyond lactation, histone acetylation also regulates metabolic processes in livestock. In weaning piglets, sodium butyrate alleviates deoxynivalenol-induced hepatic cholesterol metabolic dysfunction via RORγ-mediated histone acetylation modification [73].

#### 3.2.4. Immunity

In bovine peripheral blood mononuclear cells (PBMCs), LPS induces differential expression of HDAC6, HDAC7, and *DNMT3A* genes. Treatment with the HDAC inhibitor TSA significantly inhibits pro-inflammatory cytokine expression, suggesting an important role for HDACs in bovine innate immune regulation [74]. In pigs, butyrate upregulates endogenous host defense peptides via HDAC inhibition, thereby enhancing disease resistance against E. coli infection [75]. PRRSV infection downregulates HDAC2 expression in porcine macrophages. Suppressing HDAC2 activity increases virus production, while overexpressing HDAC2 inhibits PRRSV replication by boosting IFN-regulated antiviral molecules [76]. In chickens, p53 recruits the HDAC1/2 complex to the ALV promoter to suppress viral promoter activity and replication. Importantly, the acetylation status of ALV-bound histones H3 and H4 correlates with ALV viremia in infected chickens [77].

### 3.3. Other Histone Modifications in Economic Traits

Beyond methylation and acetylation, additional histone post-translational modifications, including phosphorylation, ubiquitination, crotonylation, and lactylation, have emerged as important regulators of economic traits in livestock.

Histone ubiquitination plays a notable role in fat deposition. In pigs, RNF20 is a key E3 ligase responsible for histone H2B monoubiquitination (H2Bub). RNF20 and H2Bub expression levels are significantly higher in backfat tissues of fat-type pigs compared to lean-type pigs. Knockdown of RNF20 suppresses porcine preadipocyte differentiation by impairing mitotic clonal expansion, indicating that the RNF20-H2Bub axis positively regulates adipogenesis in pigs [78].

Histone lactylation is a recently discovered modification that directly links cellular metabolism to epigenetic regulation. In dairy cows, a high-concentrate diet induces subacute ruminal acidosis and elevates lactic acid concentrations in both plasma and the mammary gland. This metabolic shift significantly upregulates global histone lactylation levels, particularly H3K18la, mediated by p300/CBP. The elevated lactylation then activates the TLR4/NF-κB signaling pathway, triggering inflammatory responses in the mammary gland [79]. This study demonstrates that dietary-induced metabolic changes directly influence histone lactylation, which in turn affects tissue inflammation and health. In yaks, feed restriction reduces ruminal lactate levels and histone lysine lactylation. Glutamine supplementation alleviates this reduction by increasing lactate concentrations, p300 expression, and histone lactylation levels, thereby repairing malnutrition-induced damage to rumen epithelial barrier function [80]. These findings highlight how nutritional intervention can restore metabolic balance through the regulation of histone lactylation.

Other modifications such as histone crotonylation and phosphorylation have also been detected in livestock tissues, but their functional roles in economic traits remain largely unexplored. Future studies are warranted to investigate these epigenetic marks and their potential applications in livestock breeding and health management.

### 3.4. Cross-Species Disparity and Research Gaps in Farm Animal Epigenetic Regulation

Research on histone modifications associated with economic traits varies substantially across livestock and poultry species. This disparity is evident in both research depth and trait coverage. Pigs and cattle represent the best-studied mammalian models for epigenetic research in animal husbandry. Cumulative empirical evidence has validated the regulatory roles of core histone markers in multiple crucial biological processes, including muscle development, meat quality formation, fat deposition, reproductive performance and immune response. To date, numerous functional target genes have been identified, and complete epigenetic regulatory networks have been systematically characterized. These research advances have jointly established a relatively complete theoretical foundation for epigenetic breeding in pigs and cattle.

By contrast, epigenetic research on sheep and goats remains at an early stage. Several recent multi-omics studies have made preliminary attempts to decode histone-mediated regulatory mechanisms underlying muscle development, adipose thermogenesis and genomic cis-regulatory patterns in these two species. However, existing findings are relatively fragmented and only cover a limited range of traits. Research focusing on reproductive performance, heat tolerance and disease resistance in sheep and goats is still largely absent, limiting epigenetic improvement of their economic traits.

For poultry species, existing histone modification studies are also relatively insufficient compared with mammalian livestock. Current poultry epigenetic evidence is mainly concentrated on skeletal muscle development, heat stress response and immune regulation. Most reported regulatory patterns are merely correlational, lacking in-depth mechanistic exploration, reliable verified target genes, and large-population validation. Moreover, few studies have systematically clarified the conserved or species-specific histone regulatory mechanisms between poultry and mammals under analogous economic trait backgrounds.

Overall, significant interspecific research gaps still exist in farm animal epigenetic studies. The uneven research depth across species restricts the establishment of universal epigenetic regulatory models and limits the translational application of histone markers in cross-species livestock breeding. To clearly illustrate the differences in research depth and existing gaps among species for each economic trait, a species-by-trait comparison table (Table 2) is added to summarize the research status, core histone modification sites, and current limitations of histone modification studies in pigs, cattle, sheep/goat, and poultry.

## 4. Epigenetic Strategies in Livestock Breeding

The growing understanding of histone modifications has profoundly expanded the conceptual framework of livestock breeding far beyond the boundaries of conventional DNA sequence selection. Unlike static genomic polymorphisms that remain unchanged throughout the lifespan of an animal, histone modifications represent highly dynamic regulatory states. These epigenetic marks serve as an active biological bridge connecting the native genotype with developmental plasticity and fluctuating environmental inputs. Moving these fundamental discoveries from the laboratory to the farm requires a paradigm shift. An integrative framework for designing ideal production phenotypes is needed. At the applied level, the agricultural community is now embracing a multifaceted approach. This comprehensive strategy involves utilizing epigenetic biomarkers for modern marker assisted selection, exploiting nutritional programming during critical developmental windows, and pioneering the application of precision epigenome editing.

### 4.1. Epigenetic Biomarkers for Marker-Assisted Selection

Histone modifications, as direct regulators of transcription, often reflect the functional state of genes more accurately than static DNA sequences [81,82]. Recent genome-wide profiling efforts have successfully identified specific modification signatures that govern complex agronomic traits across various species. For instance, extensive epigenetic mapping in pigs has revealed discrete H3K27ac hotspots and super enhancer regions intimately linked to adipocyte differentiation and systemic lipid metabolism [83]. Similarly, researchers investigating poultry have uncovered specific three-dimensional promoter and enhancer interactions driven by targeted histone modifications that strictly govern skeletal muscle development [84]. These functional regulatory elements serve as highly promising candidate epigenetic biomarkers for future breeding programs.

Integrating such functionally relevant markers into modern genomic selection models provides a distinct advantage over traditional methods. This integration allows breeders to improve prediction accuracy for complex traits. They can prioritize noncoding variants that actively shape gene expression networks [85]. Certain stable epigenetic modification patterns are established very early in life [86]. Leveraging these biomarkers enables early prediction of mature performance metrics. This drastically shortens generation intervals in commercial breeding programs. Despite these theoretical advantages, translating epigenetic biomarkers into routine agricultural practice requires overcoming substantial hurdles. The high cost and limited throughput of current detection technologies remain significant barriers to large-scale population screening [87]. Moreover, epigenetic marks exhibit profound tissue and developmental stage specificity. This necessitates advanced diagnostic tools capable of detecting cell-type-specific histone modifications within intact tissues. Such tools ensure accurate physiological sampling [88]. For reliable application in commercial breeding, these markers require stable transgenerational inheritance of acquired epigenetic information. This ensures that superior regulatory profiles are passed to subsequent generations [89].

### 4.2. Nutritional Programming of Ideal Phenotypes

Beyond passive genetic selection, the innate environmental sensitivity of histone modifications enables the proactive shaping of production traits through carefully targeted nutritional interventions. This strategy relies heavily on the core principles of epigenetic programming, a biological phenomenon where the early nutritional environment exerts lasting developmental effects on mature metabolism and growth trajectories [90]. The fundamental biochemical foundation of this approach lies in the fact that dietary metabolites serve as essential substrates and catalytic cofactors for chromatin modifying enzymes. One highly prevalent approach involves enriching maternal diets during late gestation with specific methyl donors such as betaine and folate. These vital nutrients supply abundant S-adenosylmethionine to the developing fetus, fundamentally optimizing H3K4me3 levels at the promoters of vital metabolic genes to enhance postnatal growth performance [91].

Short-chain fatty acids such as sodium butyrate act as natural histone deacetylase inhibitors. When provided to young animals, they are introduced directly into the biological system. This dietary intervention increases global acetylation levels across the genome. This biochemical process suppresses excessive visceral fat deposition. It also activates critical immune and systemic stress resistance networks [92]. To maximize the physiological efficacy of these interventions, precision animal nutrition must strictly target specific developmental windows. Adipogenic potential is largely programmed during early fetal development in the womb. The same is true for the initial proliferation of adipose progenitor cells [93]. In ruminants, skeletal muscle characteristics and myofiber transition can be modulated by diet. This modulation responds most robustly to specific dietary inputs during rapid postnatal growth [94]. Recognizing these distinct temporal windows is essential. It helps formulate diets that actively guide the epigenome toward optimal agricultural outputs.

### 4.3. Synergistic Integration of Epigenetic Strategies

Ultimately, maximizing the true biological potential of the livestock epigenome requires recognizing how deeply intertwined these individual molecular strategies truly are. Epigenetic marker-assisted selection serves as the foundational diagnostic step to identify individuals possessing superior intrinsic regulatory potential within a given herd. Nutritional programming then provides the highly optimized biochemical environment necessary to fully realize and sustain that inherited genetic potential throughout the entire production cycle. High-throughput detection technologies are becoming more affordable. Together, these advances will enable the integration of epigenetic strategies, providing massive momentum for sustainable and efficient genetic improvement of global livestock populations.

## 5. Conclusions and Future Perspectives

Histone modifications are critical epigenetic mediators. They respond to fluctuating environmental signals and modulate the expression of genetic information. In doing so, they fundamentally shape economically important traits in agricultural animals. This review has delineated how these dynamic regulatory marks govern key biological processes, including skeletal muscle growth, adipocyte differentiation, and reproductive performance. Accumulating multi-omics evidence underscores that stable histone modification landscapes are closely associated with phenotypic variation. This association signals a crucial conceptual transition in the field. The focus shifts from pure biological exploration toward evaluating practical applications in advanced livestock breeding programs.

Among all histone modifications, H3K4me3, H3K27me3, H3K9ac, and H3K27ac show the strongest and most consistent functional evidence in livestock and poultry. These marks have been validated in muscle development, fat deposition, reproduction, and immunity across pigs, cattle, and chickens. Currently, no histone modification marks have been validated and applied in commercial breeding programs. Several marks show potential as candidate epigenetic biomarkers, but large-population verification, cost-effective detection techniques, and transgenerational stability data remain insufficient.

In addition, tissue specificity is a major bottleneck restricting the application of histone modification markers in livestock breeding. Most histone marks exhibit tissue-specific regulatory patterns, making it difficult to obtain universal and stable biomarkers from easily sampled tissues. To address this issue, future studies are required to screen stable, tissue-consistent histone modification signatures that are uniformly expressed in peripheral accessible tissues and core functional tissues, which can effectively eliminate the interference of tissue specificity and improve the practicability of epigenetic markers in animal breeding. A profound mechanistic knowledge gap persists when comparing agricultural species to well-established model organisms. In murine and cell culture models, the intricate molecular choreography of histone writers, readers, and erasers has been dissected down to precise protein interactions and atomic structures. Conversely, epigenetic research in livestock and poultry remains predominantly descriptive and inherently correlational. Current agricultural studies rely heavily on broad genomic profiling to identify modification hotspots, yet the specific upstream signaling cascades and precise targeting mechanisms that direct these epigenetic enzymes to specific genomic loci in farm animals are still poorly understood. Consequently, the field lacks the rigorous in vivo experimental validation required to confirm whether specific histone modifications are the direct drivers of phenotypic diversity or merely the downstream consequences of other transcriptional events.

Translating this foundational knowledge into scalable breeding technologies requires a paradigm shift toward multi-dimensional, high-resolution methodologies. To disentangle the profound cellular heterogeneity inherent in complex production traits, the field must transition away from traditional bulk assays and embrace cutting edge single-cell and spatial epigenomics. Concurrently, moving beyond the linear genome to explore dynamic 3D chromatin architecture will be absolutely crucial for mapping the precise enhancer and promoter interactomes that ultimately dictate cell fate and tissue development. By comprehensively achieving dynamic multi-omics integration across epigenomic, transcriptomic, and three-dimensional structural layers, researchers can establish highly accurate functional annotations for noncoding genetic variants. This comprehensive integration directly bridges the gap between static genetic markers and true biological causality. When coupled with precision tools like CRISPR based epigenome editing for rigorous in vivo functional validation, this highly annotated regulatory blueprint will fundamentally transform genomic evaluation models. Ultimately, leveraging this multi-dimensional epigenetic framework will enable the accurate early prediction and active shaping of ideal livestock phenotypes, propelling global animal husbandry into an era of sustainable, precise, and highly efficient production.

## Figures and Tables

**Table 1:** Summary of histone modifications.

Modification Type	Primary Modification Sites	Writer	Eraser	Key Function
Methylation	Active: H3K4me1/2/3, H3K36me2/3 Repressive: H3K9me2/3, H3K27me2/3, H4K20me2/3	H3K4: KMT2 famil, SETD1A/B H3K36: SETD2 (me3), NSD family (me2) H3K9: SUV39H1/2, G9a/GLP, SETDB1 H3K27: EZH2 H4K20: SUV420H1/2 (me2/3), SET8 (me1)	H3K4: KDM5 family, LSD1 H3K36: KDM4 family, KDM2 family H3K9: KDM4 family, KDM3 family, LSD1 H3K27: UTX/KDM6A, JMJD3/KDM6B H4K20: KDM7 family etc. (less studied)	Recruit “reader” proteins containing PHD, Chromo, or Tudor domains; maintain histone charge unchanged; transmit signals via effector proteins
Acetylation	H3K9ac, H3K14ac, H3K27ac, H4K5ac, H4K12ac, H4K16ac	p300/CBP, GCN5/PCAF, GNAT, MYST	HDACs (Class I–IV), Sirtuins (Class III)	Neutralize lysine positive charge, reduce histone-DNA electrostatic interaction, leading to open chromatin; recruit bromodomain-containing transcriptional activators.
Phosphorylation	H3S10ph, H3S28ph, H3T3ph, γH2AX (H2AXS139ph), etc.	Mitosis: Aurora B DNA damage: DNA-PK, ATM/ATR Signaling: MAPK, CDK, etc.	PP1, PP2A, etc.	Introduce negative charges to alter chromatin conformation; act as signaling tags to recruit downstream effectors
Lactylation	H3K18la, H4K12la, H3K9la, H3K14la, H3K23la, H2AK9la	p300/CBP	HDAC1-3, SIRT1-3	Link metabolism to epigenetics; neutralize lysine charge; recruit effector proteins; drive 3D genome remodeling
Ubiquitination	H2BK120ub1, H2AK119ub1, H3K14ub, H2AK13/15ub	H2Bub1: RNF20/40 H2Aub1: RING1B (core PRC1) DNA damage: RNF168, etc.	Multiple DUBs: USP family, BAP1, MYSM1, BRCC36, etc.	Serve as scaffolds to recruit downstream effectors; cross-regulate with other modifications; influence DNA double-strand break repair pathway choice
SUMOylation	H2AK119su, H2BK120su, H3K4su, H3K9su, H4K5su/H4K12su	E1: SAE1/SAE2 E2: Ubc9 E3: PIAS family, RanBP2, Pc2	SENP family proteases (SENP1/2/3/5/6/7)	Predominantly associated with transcriptional repression; regulates chromatin dynamics, DNA damage repair and cell cycle progression; extensive crosstalk with histone ubiquitination and acetylation

**Table 2:** Research status of histone modifications for livestock economic traits.

Trait Category	Economic Trait	Pig	Cattle	Sheep/Goat	Poultry	Core Histone Marks	Research Gaps
Growth and Meat Quality	Skeletal muscle development	Strong	Strong	Moderate	Moderate	H3K4me3, H3K27me3, H3K9ac, H3K27ac	Sheep/Goat: lack of systematic regulatory networks; Poultry: insufficient mechanistic validation
	Fat deposition/intramuscular fat	Strong	Strong	Moderate	Limited	H3K27me3, H3K4me3, H2Bub1	Sheep/Goat: understudied in reproduction and stress resilience; Poultry: fat metabolism poorly understood
Reproduction	Male spermatogenesis/fertility	Strong	Strong	Moderate	Limited	H3K27me3, H3K9me3, H3K9ac	Sheep/Goat: reproductive epigenetics largely unexplored; Poultry: male epigenetic regulation unclear
	Female oocyte/embryo development	Strong	Strong	Limited	Limited	H3K4me3, H3K27me3, H3K27ac	All species: lack of validated transgenerational stability
Lactation and Metabolism	Lactation performance	Limited	Strong	Limited	Limited	Histone acetylation, lactylation	Well-studied only in cattle; largely lacking in other species
	Metabolic health	Moderate	Strong	Limited	Moderate	H3K27me3, histone lactylation	Cross-species conserved mechanisms remain elusive
Environment and Immunity	Heat stress adaptation	Limited	Limited	Limited	Moderate	H3K4me3, H3K27me3	All species: lack of systematic epigenetic research on heat stress
	Disease resistance/immunity	Moderate	Strong	Limited	Moderate	H3K4me3, H3K27me3	Sheep/Goat: immune epigenetic regulation remains largely uncharacterized

Research intensity is classified into three grades. Strong indicates sufficient evidence with well-elucidated regulatory mechanisms and validated target genes. Moderate represents preliminary but fragmented findings with partial mechanistic exploration. Limited denotes scarce or absent epigenetic evidence for corresponding economic traits.

## Data Availability

No new data were created or analyzed in this study.

## Abbreviations

The following abbreviations are used in this manuscript:

GS	Genomic selection
SNP	Single nucleotide polymorphism
HMTs	Histone methyltransferases
KDMs	Histone demethylases
SAM	S-adenosylmethionine
PRC2	Polycomb repressive complex 2
HATs	Histone acetyltransferases
HDACs	Histone deacetylases
DUBs	Deubiquitinating enzymes
SSCs	Spermatogonial stem cells
SCNT	Somatic cell nuclear transfer
ZGA	Zygotic genome activation
FLHS	Fatty liver hemorrhagic syndrome
HSPs	Heat shock proteins
MDV	Marek’s disease virus
KAT5	Lysine acetyltransferase 5
LPS	Lipopolysaccharide
PBMCs	Peripheral blood mononuclear cells
me	Methylation
ac	Acetylation
ph	Phosphorylation
ub	Ubiquitination
la	Lactylation
